# Comorbid generalized anxiety disorder and its association with quality of life in patients with major depressive disorder

**DOI:** 10.1038/srep40511

**Published:** 2017-01-18

**Authors:** Yongjie Zhou, Zhongqiang Cao, Mei Yang, Xiaoyan Xi, Yiyang Guo, Maosheng Fang, Lijuan Cheng, Yukai Du

**Affiliations:** 1 Department of Maternal and Child Health, School of Public Health, Tongji Medical College, Huazhong University of Science & Technology, HangKong Road 13, Wuhan, 430030, China; 2 Affiliated Mental Health Center, Tongji Medical College of Huazhong University of Science & Technology, GongNongBin Road 125#, Wuhan, 430012, China; 3 Affiliated Liyuan hospital, Tongji Medical College of Huazhong University of Science & Technology, YuanHu Road 39#, Wuhan, 430060, China

## Abstract

The comorbidity of major depressive disorder (MDD) and generalized anxiety disorder (GAD) is common and often predicts poorer outcomes than either disorder alone. This study aimed to examine the prevalence of comorbid GAD and its association with quality of life (QOL) among MDD patients. A total of 1225 psychiatric outpatients were screened using the Hospital Anxiety and Depression Scale (HADS). Those who scored ≥8 on the HADS were interviewed using DSM-IV criteria by two senior psychiatrists. Patients diagnosed with MDD were further assessed using the 9-item Patient Health Questionnaire, Social Support Rating Scale, Pittsburgh Sleep Quality Index, and World Health Organization QOL Scale, brief version (WHOQOL-BREF). Ultimately, 667 patients were diagnosed with MDD, of 71.7% of whom had GAD. Compared to those with MDD alone, comorbid patients had lower scores on the physical (38.64 ± 10.35 vs.36.54 ± 12.32, *P* = 0.026) and psychological (35.54 ± 12.98 vs. 30.61 ± 14.66, *P* < 0.001) domains of the WHOQOL-BREF. The association between comorbid GAD and poor QOL on the two domains remained statistically significant in the multiple linear regression (unstandardized coefficients: −1.97 and −4.65, *P* < 0.001). In conclusion, the prevalence of comorbid GAD in MDD patients is high, and co-occurring GAD may exacerbate impaired physical and psychological QOL in Chinese MDD patients.

Comorbidity of depression and anxiety is common in health care settings^1^,^2^, and this phenomenon has attracted a great deal of research and clinical attention^3^. Major depressive disorder (MDD), the most severe sub-type of depressive disorder, also has a high rate of comorbidity with other psychiatric illnesses in clinical samples^2^. In the US National Comorbidity Survey, the prevalence of co-occurrence with other psychiatric disorders and any type of anxiety disorder in MDD patients is 76.7% and 56.8%, respectively^4^. A recent empirical study showed that more than 80% of patients with MDD or bipolar disorder have at least one current comorbid mental disorder^2^. Epidemiological data also suggest that 59.0% of individuals with generalized anxiety disorder (GAD) meet the criteria for MDD^5^. Therefore, comorbid MDD and GAD is the most common form of comorbidity involving depression and anxiety^2^,^6^,^7^. Although the prevalence of co-occurring GAD among MDD patients varies across clinical samples, comorbid MDD and GAD is definitely a significant clinical concern associated with individuals who are receiving psychiatric treatment.

In Western countries, studies have found that, compared to patients with MDD alone, comorbidity of MDD and anxiety disorder is strongly associated with a poorer prognosis^8^, more severe symptoms^7^,^9^, more serious role impairment^4^, earlier age of initial onset of MDD^7^, poorer quality of life (QOL)^8^,^10^,^11^, greater MDD recurrence^12^ and higher suicide risk^13^,^14^. In China, most available comorbidity studies in this field focus on the comorbidity of physical disease, such as cancer, diabetes mellitus, and cardio-cerebrovascular diseases with depression and anxiety, rather than comorbidity with mental disorders. Only a few Chinese studies have investigated the prevalence and clinical characteristics of comorbid depressive disorder and anxiety disorder^15^,^16^. However, they primarily focused on non-clinical samples only (i.e., older adults and medical students). To the best of our knowledge, no research has examined the impact of comorbid GAD on the QOL of Chinese MDD patients in clinical settings.

Since the end of the 20th century, QOL has been a key outcome in the planning and evaluation of health services, including the assessment of disease burden and monitoring of treatment effectiveness^17^,^18^. Mental disorders significantly contribute to diminished QOL^19^,^20^. Both cross-sectional and longitudinal studies have documented the significant association between the presence of psychiatric disorders and decreased QOL^19^,^21^. More importantly, studies have found that, among the various mental disorders, MDD is associated with the largest reduction in QOL^22^. Research has indicated that MDD patients’ QOL is poorer than that of patients with physical disease^23^. Further, it has been well-established that co-existing depression and anxiety poses the greatest threat to QOL^10^,^24^. However, current evidence relating to the association between MDD and comorbid anxiety disorder with poor QOL has almost exclusively been derived from research conducted in developed countries. Few studies have explored the influence of MDD with comorbid anxiety disorder on patients’ QOL in China. Therefore, it is imperative to examine this situation in Chinese patients with MDD and investigate the impact of comorbidity on QOL to improve treatment outcomes, as well as the prognosis of this patient population.

In the literature, many factors have been found to be associated with the QOL of psychiatric patients, including age, gender, marital status, acquired social support, symptom severity, comorbidity, remission and residual symptoms, and sleep quality^25^,^26^,^27^,^28^,^29^,^30^. There is evidence that GAD is also associated with poor QOL^19^,^31^; however, whether such a comorbid condition is independently associated with the QOL of MDD patients remains unclear. We hypothesize that coexisting GAD is a significant and independent contributing factor to the reduced QOL of MDD patients. The present study investigated the prevalence and socio-demographic characteristics of comorbid GAD in Chinese outpatients with MDD, and examined the association of such comorbidity with their QOL.

## Results

### Participant characteristics

A total of 667 patients diagnosed with MDD were included in this study. Their mean age was 31.0 years (standard deviation = 10.9, range 18–71). Additionally, 54.9% of participants were female (n = 366) and 7.9% (n = 53) were divorced/separated/widowed. Fifteen percent of patients (n = 100) had an educational level of junior high school or lower. Participants’ socio-demographic characteristics are displayed in Table 1.

### Prevalence and characteristics of comorbid GAD in MDD patients

A total of 478 patients were diagnosed with GAD. The prevalence of comorbid GAD in the patients with MDD was 71.7%. The results of comparisons between MDD patients with and without GAD (Table 2) showed that patients with comorbid GAD were more likely to be female, have a marital status of “other” (single, divorced, widowed, separated), be depressed, have poor physical and psychological QOL, have less objective support, and have poor sleep quality.

### QOL of MDD patients with and without GAD

Table 2 also shows that MDD patients with GAD had significantly lower physical (36.54 ± 12.32 vs. 38.64 ± 10.35, *P* = 0.026) and psychological (30.61 ± 14.66 vs. 35.54 ± 12.98, *P* < 0.001) QOL scores than those without GAD.

### Univariate and multivariate analyses involving factors related to physical QOL

Independent sample *t*-tests showed that MDD patients who were unemployed, had a marital status of “other”, had a low level of subjective social support (subjective support score <17), had lower objective social support (objective support score <7) and utilization of social support (utilization score <7), had poor sleep quality (PSQI ≥ 7) and suffered from GAD had poor physical QOL (*t* = 3.89–7.97, *P* < 0.05).

After entering all the variables that were significantly associated with physical QOL into the multiple linear regression model, we found that comorbid GAD was still significantly associated with poor physical QOL (*β* = − 1.97, *P* < 0.001) (Table 3).

### Univariate and multivariate analyses on involving factors related to psychological QOL

Independent sample *t*-tests showed that MDD patients who were unemployed, had a marital status of “other”, had a low level of subjective social support (subjective support score <17) and utilization of social support (utilization score <7), had poor sleep quality (PSQI ≥ 7) and suffered from GAD had poor psychological QOL (*t* = 3.64–8.64, *P* < 0.05).

After entering all the variables that were significantly associated with psychological QOL into the multiple linear regression model, we found that comorbid GAD was still significantly associated with poor psychological QOL (*β* = −4.65, *P* < 0.001) (Table 4).

## Discussion

The present study examined the clinical epidemiology of GAD and its potential effect on the QOL of patients with MDD. We found that (1) the prevalence of comorbidity of MDD and GAD was high in Chinese MDD patients, (2) MDD patients with GAD had more depressive symptoms, reduced sleep quality and poorer physical and psychological QOL compared to patients with MDD only, and (3) co-occurring MDD and GAD was still significantly associated with lower physical and psychological QOL, after adjusting for potential confounders. These findings indicate that GAD is a common clinical issue for MDD patients, that may further cause the deterioration of depressive symptoms, sleep quality and physical and psychological QOL. It is important to note that the association between comorbid GAD and QOL remained significant in multiple regression analyses, suggesting that comorbid GAD might be a robust predictor of diminished QOL among MDD patients.

Compared to Western studies involving MDD patients^10^,^19^,^32^,^33^, the 71.7% prevalence of comorbid GAD that we found in this study was higher. Our prevalence estimate was also higher than the one reported in a Chinese study by Gao *et al*.^34^, which reported that more than 80% of patients with MDD or bipolar disorder had at least one current comorbid disorder. However, the prevalence of the most common type of comorbidity, “MDD + AD”, was only 58.4%. These variations in prevalence could be ascribed to disparities in clinical settings (i.e., outpatient vs. inpatient), sampling methods (convenient vs. consecutive sampling), racial differences (Chinese vs. Caucasian) and the overlap between depressive and anxiety symptomatology (insomnia). Nevertheless, because our subjects were recruited from a psychiatric specialty hospital and only those who met the criteria based on their scores on the Hospital Anxiety and Depression Scale (HADS) were eligible for the inclusion of this study. We believe that the unique research setting and screening tool used in this study are two of the main explanations for this high prevalence.

The high prevalence of GAD in MDD patients could be explained by the common neurobiological mechanism underlying anxiety and depression. Laboratory studies have shown that the dysfunction of serotoninergic, noradrenergic and dopaminergic neurotransmission; abnormal regulation in the hypothalamic-pituitary-adrenal axis; disturbance of cellular plasticity (i.e., reduced neurogenesis); and chronic inflammation connected with high oxidation are all involved in the development of anxiety and depression^35^,^36^.

Numerous studies have provided evidence that mental disorders, particularly depression and anxiety, significantly diminish QOL^17^,^19^,^24^,^37^,^38^,^39^. Our findings were in line with those of previous studies. In this study, the association of comorbid MDD and GAD and poor QOL was independent of socio-demographic factors, social support and sleep quality, which indicates that the effect of comorbid GAD on QOL prevails over these factors and, perhaps, that GAD impairs QOL via a mechanism other than reduced social support and sleep quality. Still, it is possible that GAD directly leads to poorer psychological QOL, given that psychological QOL can be viewed as a comprehensive measure of mental health.

Several limitations should be considered when interpreting the findings of the present study. First, although many common influential factors were assessed in the study, data on medical conditions, treatment response, and life events that may also negatively impact QOL were not collected and may have confounded our findings. Second, the cross-sectional data in this study made it impossible to infer causality in the association between comorbid MDD and GAD and QOL. Third, because QOL was measured subjectively, it is possible that the negative emotions of MDD patients biased their perception of actual QOL. Fourth, the selection of the study sample from a specialty psychiatric hospital also limits the generalizability of our findings. Finally, MDD patients also have other types of co-occurring mental disorders, that have negative effects on their QOL. However, we only collected data on GAD. More studies are needed to clarify other potential psychosocial risk factors associated with reduced QOL and the comorbidity profiles of MDD patients.

In summary, GAD is prevalent among MDD patients, and comorbid GAD may be a factor that significantly contributes to poor QOL in MDD patients. Given the high disease burden of MDD and the clinical relevance of comorbid GAD, it is important to routinely assess anxiety disorder and provide appropriate treatment to improve the QOL of patients with MDD.

## Methods

### Study design and participants

The study design was a two-stage cross-sectional survey. Consecutive participants were recruited from the out-patient department at Wuhan Mental Health Center. This hospital is the largest psychiatric specialty hospital in central China. It has more than 1000 inpatient beds and provides mental health services for over 10 million residents. In the first stage, patients with potential mood disorders were screened using the HADS. Those who scored ≥8 on the HADS were invited to participate in the second stage, which involved a diagnostic interview.

The study was conducted between April 2013 and April 2015. The study protocol was approved by the ethics committee of Wuhan Mental Health Center before the formal study began, and all participants provided their informed consent. The study was conducted in accordance with the guidelines of the Declaration of Helsinki and its amendments.

The inclusion criteria used to target patients were: (1) aged 18–75 years, (2) meet the diagnostic criteria in the Diagnostic and Statistical Manual of Mental Disorders Fourth Edition (DSM–IV) for MDD, (3) agreed to participate in the study and had a HADS score ≥8, and (4) had at least a primary school level of educational. The exclusion criteria included: (1) aged fewer than 18 years or more than 75 years, (2) had bipolar disorder, schizophrenia or other psychotic disorder; alcohol dependence; or severe cognitive disorder or neurological disease, or (3) refused to participate in the study.

Finally, 1225 participants agreed to part in the study and 1024 completed the first stage involving the HADS screening, which was administered by four trained psychiatric nurses. Of the 1024 completers, 685 were diagnosed with MDD. However, 18 did not provide complete data on their socio-demographic characteristics, leaving 667 participants in the sample.

### Diagnostic assessment

Participants were interviewed about their psychiatric diagnoses by two senior psychiatrists with at least 10- years of clinical experience. They also received extensive training on how to use the DSM-IV.

### Assessment instrument

In the study questionnaire, subjects’ socio-demographic variables included gender, age, marital status, education level, work status and economic status. Data on other clinically relevant variables were collected. Information on clinical variables was collected using the following instrument:

### Hospital Anxiety and Depressive Disorders Rating Scale (HADS)

The HADS consists of seven items relating to anxiety (HADS-A) and seven items relating to depression (HADS-D). The items were scored on a 4–point scale, with responses ranging from zero (not present) to 3 (considerable)^40^. Higher scores represent higher symptom levels. Additionally, a score of 8 was considered to be an optimal cut-off value to identify respondents with potential anxiety or depression. The Chinese version of the HADS (C-HADS) has good internal consistency and test-retest reliability, with a Cronbach’s coefficient alpha of 0.85 and intra-class correlation coefficient of 0.90, respectively^41^. In this study, the HADS was used as a screening tool.

### Patient Health Questionnaire (PHQ-9)

The PHQ-9 consists of 9-items relating to depressive symptoms that correspond with the MDD diagnostic criteria in the DSM-IV. As a severity measure, the PHQ-9 score can range from 0 to 27. Each item score ranged from 0 (not at all) to 3 (nearly every day), which was used to evaluate the severity of depressive symptoms in our study^42^.

### World Health Organization Quality of Life Scale, Brief Version (WHOQOL-BREF)

The WHO QOL-BREF is a short version of the WHOQOL-100 scale. It contains 26-items, and includes four domains, including physical health, psychological health, social relationships and environment^43^. Assessments were conducted over the preceding 2 weeks. The response options ranged from 1 (very dissatisfied/very poor) to 5 (very satisfied/very good). Each of the four domains was scored on a scale with a maximum score of 100. Higher scores indicate higher QOL. The internal consistency (Cronbach’s alpha) value for the entire population of subjects was 0.93. The psychometric properties (reliability and validity) of the WHOQOL-BREF have been shown to be highly satisfactory in patients with psychiatric disorders^44^. The instrument has been applied in various clinical studies and general population surveys of the Chinese population and exhibited good test-retest reliability^45^. In the study, we employed the physical and psychological domains of the WHOQOL-BREF only.

### Social Support Rating Scale (SSRS)

The Chinese version of the SSRS includes a Likert scale, developed by Xiao ShuiYuan in 1994 and is used to assess individuals’s social support status. The SSRS consists of 10 items and three dimensions, namely objective support (3 items), subjective support (4 items), and support utilization (3 items)^46^. Each item score ranges from 1 to 4 (1 = none, 2 = slight, 3 = moderate, 4 = great). Higher scores indicate better social support. This tool has been found to have good reliability and validity. Cronbach’s alpha coefficients for the total scale and subscales ranged from 0.825 to 0.896^47^. In this study, we used the median split approach to define a subject’s level of social support, given that there are no recommended cut-off values for the SSRS.

### Pittsburgh Sleep Quality Index (PSQI)

The PSQI is a 19-item questionnaire based on an assessment of sleep patterns that measures subjective sleep quality during the previous month. It had been widely used in clinical and epidemiological studies to monitor and evaluate subjective sleep quality in healthy people and people with psychiatric and medical disorders^48^,^49^. The PSQI consists of seven components: subjective sleep quality, sleep latency, sleep duration, habitual sleep efficiency, sleep disturbances, use of hypnotic drugs, and daytime dysfunction. Global PSQI scores range from 0–21, with higher scores represent poorer sleep quality. A global PSQI score ≥7 had been recommended to distinguish poor sleepers from good sleepers^50^. The Chinese version of the PSQI (CPSQI) reportedly has a coefficient of 0.85 for all subjects and 0.77 for people with primary insomnia^51^.

### Statistical Analyses

Statistical analyses were performed using SPSS version 18.0 (SPSS Inc., Chicago, Illinois, USA). The two-tailed statistical significance level was set to at 0.05. Descriptive analyses were conducted to describe the demographic characteristics and prevalence of comorbid GAD among MDD patients. Chi-square tests were used to investigate the differences on categorical variables. Two-tailed Student’s *t*-tests were used to examine the differences between the two groups. The univariate analyses was performed by comparing the QOL scores between patients based on their socio-demographic and clinical characteristics (i.e., male vs. female, poor sleep quality vs. good sleep quality) using Student’s *t*-tests.

Multiple linear regression analyses were employed to test the independent association between comorbid GAD and QOL. The physical and psychological domains QOL were used as dependent variable, and demographic factors, social support and sleep quality were used as the independent variables. All variables that were significantly correlated with QOL in the univariate analyses were included in the multiple regression model simultaneously.

## Additional Information

**How to cite this article**: Zhou, Y. *et al*. Comorbid generalized anxiety disorder and its association with quality of life in patients with major depressive disorder. *Sci. Rep.* **7**, 40511; doi: 10.1038/srep40511 (2017).

**Publisher's note:** Springer Nature remains neutral with regard to jurisdictional claims in published maps and institutional affiliations.

## Figures and Tables

**Table 1:** Socio-demographic characteristics of the study samples (*N* = 667).

Characteristics	Values
Age, *n* (%)	
18~30 years	389 (58.3)
31~40 years	142 (21.3)
41~50 years	83 (12.4)
51~60 years	43 (6.5)
61~71 years	10 (1.5)
Age (years), mean (SD)	31.0 (10.9)
Gender, *n* (%)	
Male	301 (45.1)
Female	366 (54.9)
Marital status, *n* (%)	
Married or cohabiting	296 (44.4)
Single	318 (47.7)
divorced or separated	41 (6.1)
widowed	12 (1.8)
Educational level, *n* (%)	
<9 years	100 (15.0)
9–12 years	188 (28.2)
>12 years	379 (56.8)
Employment status, *n* (%)	
Employed	439 (65.8)
Unemployed or demission	228 (34.2)
Monthly family income (RMB), *n* (%)	
0–3000	227 (34.0)
3001–10000	373 (56.0)
≥10001	67 (10.0)

**Table 2:** Comparisons of socio-demographic characteristics, quality of life, social support and sleep quality between MDD with GAD and without GAD.

Various factor	MDD only (N = 189)	MDD + GAD (N = 478)	*χ*^*2*^	*P* value
	n	n		
Gender				
male	126	175	49.41	<0.001
female	63	303		
Educational level				
≤9 years	33	67	2.80	0.246
9–12 years	58	130		
>12 years	98	281		
Marital status				
Married or cohabiting	82	214	14.64	<0.001
Others*	107	264		
Employment status				
Employed	70	164	0.44	0.529
Unemployed or demission	119	314		
Monthly family income (RMB)				
0–3000	60	167	0.85	0.654
3001–10000	111	262		
≥10001	18	49		
	**Mean ± SD**	**Mean ± SD**	***t***	***P*** **value**
Age (years)	30.60 ± 10.75	31.21 ± 11.00	0.65	0.516
PHQ-9 score	16.52 ± 3.28	18.92 ± 4.00	7.33	＜0.001
WHOQOL-BREF scale score				
Physical domain	38.64 ± 10.35	36.54 ± 12.32	2.23	0.026
Psychological domain	35.54 ± 12.98	30.61 ± 14.66	4.04	<0.001
Social Support Rating Scale				
Subjective support	17.71 ± 4.23	17.55 ± 4.44	0.43	0.671
Objective support	7.32 ± 2.53	6.56 ± 2.82	3.22	0.001
Utilization degree	6.74 ± 1.95	6.66 ± 1.97	0.47	0.636
PSQI score	9.07 ± 5.13	9.99 ± 5.32	27.16	<0.001

^*^Others included single, divorced, separated and widowed. Chi-square test was used to test the difference of sociodemographic characteristic between MDD patients with D and without GAD, except for age. Students *t*-test was used to test the difference of age, WHOQOL-BRIEF, SSRS and PSQI scores between patients with MDD comorbid GAD and MDD alone, which presented as the means ± SD, and SD = Standard deviation. The statistical significance level was set to a two-tailed 0.05.

**Table 3:** Multiple linear regression analyses on the relationship between comorbid GAD and physical QOL.

Associated factor	Unstandardized coefficient	Standard error	Standardized coefficient	*t*	*P* value
Marital status of “others”	−1.89	0.71	−0.11	2.66	0.008
Unemployment	−0.21	0.16	−0.05	1.32	0.189
Social Support Rating Scale					
Subjective support score <17	−0.27	0.12	0.10	2.17	0.030
Objective support score <7	−0.05	0.19	0.01	0.24	0.813
Utilization of support <7	−0.68	0.24	0.11	2.79	0.005
Poor sleep quality	−1.26	0.64	−0.09	1.98	0.048
Comorbid GAD	−1.97	0.99	−0.08	1.99	0.047

“Others” included single, divorced, separated and widowed.

**Table 4:** Multiple linear regression analyses on the relationship between comorbid GAD and psychological QOL.

Associated factor	Unstandardized coefficient	Standard error	Standardized coefficient	*t*	*P* value
Marital status of “others”	−1.01	0.85	−0.05	1.18	0.238
Unemployment	−0.29	0.19	−0.06	1.49	0.137
Social Support Rating Scale					
Subjective support score <17	−0.41	0.15	0.13	2.75	0.006
Utilization of support <7	−0.85	0.29	0.12	2.88	0.004
Poor sleep quality	−1.91	0.68	−0.11	2.81	0.005
Comorbid GAD	−4.65	1.19	−0.15	3.90	<0.001

“Others” included single, divorced, separated and widowed.
